# Supplementary material for: Effect of Clindamycin on Intestinal Microbiome and Miltefosine Pharmacology in Hamsters Infected with Leishmania infantum
Source: Antibiotics (Basel). 2023 Feb 9;12(2):362. doi: 10.3390/antibiotics12020362 (PMC9952363; doi:10.3390/antibiotics12020362)
Supplement: Supplementary file 1 [file antibiotics-12-00362-s001.zip › 1.SUPPLEMENTARY MATERIAL 1 FIGURE S1.pdf]

SUPPLEMENTARY MATERIAL 1 (Figure S1)

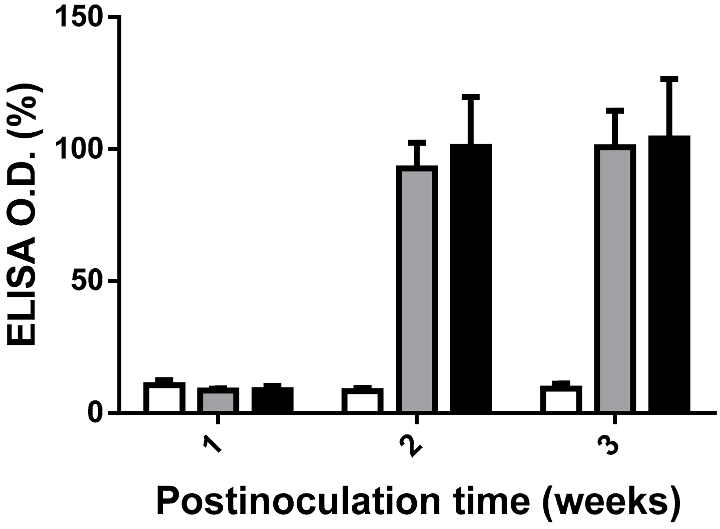

Figure S1. Serum-specific anti-*Leishmania infantum* IgG response of *L.infantum*-inoculated and control hamsters throughout the experiment determined by ELISA. Empty bars: Uninfected animals; Grey bars: infected and treated with miltefosine; Solid bars: infected and treated with miltefosine and clindamycin. ELISA values correspond to the % of positive control serum. Values are mean ± standard deviation.
