# Supplementary material for: Effect of Clindamycin on Intestinal Microbiome and Miltefosine Pharmacology in Hamsters Infected with Leishmania infantum
Source: Antibiotics (Basel). 2023 Feb 9;12(2):362. doi: 10.3390/antibiotics12020362 (PMC9952363; doi:10.3390/antibiotics12020362)
Supplement: Supplementary file 1 [file antibiotics-12-00362-s001.zip › 2.SUPPLEMENTARY MATERIAL 2 Tables S1a, S1b and S1c (rev).pdf]

SUPPLEMENTARY MATERIAL 2. Table S1a; Table S1b and Table S1c

Table S1a: Phyla abundance (%) in groups G2, G4 and G5 at week 16.

Phylum	G2 16w Abundance	G4 16w Abundance	G5 16w Abundance
Firmicutes	61.1140%	58.9661%	60.5420%
Bacteroidetes	34.3401%	36.1905%	35.6117%
Proteobacteria	1.3200%	1.1671%	1.3143%
Verrucomicrobia	1.0651%	1.1755%	0.3401%
Patescibacteria	0.6014%	0.5012%	0.4671%
Cyanobacteria	0.5318%	0.8072%	0.8060%
Epsilonbacteraeota	0.4986%	0.4881%	0.4610%
Deferribacteres	0.2077%	0.1443%	0.1117%
Tenericutes	0.1619%	0.3702%	0.1901%
Actinobacteria	0.1237%	0.1276%	0.0840%
Elusimicrobia	0.0294%	0.0507%	0.0685%
Chloroflexi	0.0023%	0.0021%	0.0005%
Planctomycetes	0.0015%	0.0030%	0.0010%
Lentisphaerae	0.0008%	0.0019%	0.0012%
Deinococcus-Thermus	0.0008%	0.0008%	0.0002%
Fusobacteria	0.0005%	0.0017%	0.0005%
Spirochaetes	0.0003%	0.0004%	0.0000%
WPS-2	0.0003%	0.0004%	0.0000%
Synergistetes	0.0000%	0.0006%	0.0002%
Euryarchaeota	0.0000%	0.0002%	0.0000%
Gemmatimonadetes	0.0000%	0.0004%	0.0000%

Table S1b. Abundance (%) of the 25 most represented families in groups G2, G4 and G5 at week 16.

**Family**	**G2 16w Abundance**	**G4 16w Abundance**	**G5 16w Abundance**
Ruminococcaceae	32.2645%	32.2150%	31.4767%
Muribaculaceae	26.1638%	25.0165%	28.0887%
Lachnospiraceae	26.7086%	25.1470%	27.3458%
Prevotellaceae	5.5302%	6.1110%	4.2255%
Rikenellaceae	1.1679%	1.5924%	1.1451%
Marinifilaceae	0.7895%	2.2631%	0.8465%
Desulfovibrionaceae	1.1578%	0.9331%	1.2065%
Lactobacillaceae	1.4827%	0.4334%	0.7164%
Akkermansiaceae	1.0615%	1.1690%	0.3382%
uncultured bacterium-04	0.5132%	0.7858%	0.7921%
Peptococcaceae	0.1431%	0.1327%	0.2316%
Deferribacteraceae	0.2077%	0.1443%	0.1117%
Erysipelotrichaceae	0.1483%	0.1846%	0.0607%
uncultured-04	0.0845%	0.1204%	0.0651%
Family XIII	0.0847%	0.0956%	0.0784%
Christensenellaceae	0.0347%	0.0790%	0.0573%
Bifidobacteriaceae	0.0757%	0.0394%	0.0393%
Elusimicrobiaceae	0.0294%	0.0507%	0.0685%
Eggerthellaceae	0.0274%	0.0530%	0.0369%

Table S1c. Abundance (%) of the 25 most represented genera in groups G2, G4 and G5 at week 16.

**Genus**	**G2 16w Abundance**	**G4 16w Abundance**	**G5 16w Abundance**
uncultured bacterium-07	24.3150%	23.3357%	26.5622%
Lachnospiraceae NK4A136 group	11.9858%	8.3200%	9.2022%
uncultured-15	8.0207%	6.6331%	8.5004%
Ruminococcaceae UCG-014	6.1045%	9.8599%	6.2366%
uncultured-13	6.0374%	7.3492%	5.7860%
Ruminiclostridium 6	4.4788%	2.7187%	3.9563%
Alloprevotella	3.3918%	3.7775%	2.4781%
Ruminococcus 1	3.6513%	2.9065%	2.6891%
[*Eubacterium*] *ruminantium* group	1.0158%	3.2178%	4.5707%
[*Eubacterium*] *xylanophilum* group	2.3961%	2.4045%	2.3492%
Ruminiclostridium	2.2294%	2.3297%	2.2727%
Prevotellaceae UCG-001	2.0700%	2.2529%	1.6777%
Oscillibacter	1.3509%	2.0529%	2.1977%
Ruminiclostridium 9	1.6275%	1.8435%	1.4864%
Coprococcus 2	1.5672%	1.2512%	0.9674%
Alistipes	1.0967%	1.5048%	1.0648%
Muribaculum	1.3713%	1.1374%	1.0453%
Odoribacter	0.4991%	2.0493%	0.6028%
uncultured-26	1.0339%	0.8443%	1.1085%
Lactobacillus	1.4827%	0.4334%	0.7164%
Akkermansia	1.0615%	1.1690%	0.3382%
Ruminococcaceae UCG-003	0.7525%	0.8479%	0.9565%
Anaerosporobacter	0.1742%	0.6402%	1.7251%
uncultured bacterium-19	0.5132%	0.7858%	0.7921%
CAG-352	1.5365%	0.3707%	0.0097%
