# Supplementary material for: Effect of Clindamycin on Intestinal Microbiome and Miltefosine Pharmacology in Hamsters Infected with Leishmania infantum
Source: Antibiotics (Basel). 2023 Feb 9;12(2):362. doi: 10.3390/antibiotics12020362 (PMC9952363; doi:10.3390/antibiotics12020362)
Supplement: Supplementary file 1 [file antibiotics-12-00362-s001.zip › 3. SUPPLEMENTARY MATERIAL 3 Tables S2a, S2b and S2c (rev).pdf]

SUPPLEMENTARY MATERIAL 3. Table S2a; Table S2b and Table S2c

Table S2a: Phyla abundance (%) in groups G2 and G4 at weeks 16 and 18.

**Phylum**	**G2 16w Abundance**	**G2 18w Abundance**	**G4 16w Abundance**	**G4 18w Abundance**
Firmicutes	61.1140%	58.2260%	58.9661%	58.7055%
Bacteroidetes	34.3401%	36.0484%	36.1905%	35.7798%
Proteobacteria	1.3200%	1.7405%	1.1671%	1.9341%
Patescibacteria	0.6014%	1.7307%	0.5012%	0.8224%
Verrucomicrobia	1.0651%	0.4145%	1.1755%	0.5480%
Cyanobacteria	0.5318%	0.2992%	0.8072%	0.8523%
Epsilonbacteraeota	0.4986%	0.8571%	0.4881%	0.6056%
Tenericutes	0.1619%	0.2333%	0.3702%	0.3195%
Deferribacteres	0.2077%	0.2569%	0.1443%	0.2222%
Actinobacteria	0.1237%	0.1745%	0.1276%	0.1484%
Elusimicrobia	0.0294%	0.0127%	0.0507%	0.0497%
Chloroflexi	0.0023%	0.0011%	0.0021%	0.0033%
Planctomycetes	0.0015%	0.0000%	0.0030%	0.0031%
Fusobacteria	0.0005%	0.0022%	0.0017%	0.0031%
Lentisphaerae	0.0008%	0.0022%	0.0019%	0.0015%
Deinococcus-Thermus	0.0008%	0.0000%	0.0008%	0.0004%
Spirochaetes	0.0003%	0.0000%	0.0004%	0.0004%
Synergistetes	0.0000%	0.0004%	0.0006%	0.0000%
WPS-2	0.0003%	0.0000%	0.0004%	0.0002%
Euryarchaeota	0.0000%	0.0002%	0.0002%	0.0004%
Gemmatimonadetes	0.0000%	0.0000%	0.0004%	0.0002%

Table S2b. Differential Abundance (%) Analysis of the 25 most abundant families detected in groups G2 and G4 at weeks 16 and 18.

**Family**	**G2 16w Abundance**	**G2 18w Abundance**	**G2 18w vs G2 16w - Presence Fold Change**	**G2 18w vs G2 16w - FDR p-value**	**G4 16w Abundance**	**G4 18w Abundance**	**G4 18w vs G4 16w Log2 Presence Fold Change**	**G4 18w vs G4 16w FDR p-value**
Ruminococcaceae	32.2645%	32.5057%	1.52	0.9216	32.2150%	29.1282%	-1.08	0.9627
Muribaculaceae	26.1638%	27.7406%	1.17	0.9216	25.0165%	26.7692%	1.06	0.9627
Lachnospiraceae	26.7086%	23.1744%	1.68	0.9216	25.1470%	27.7307%	1.17	0.9627
Prevotellaceae	5.5302%	4.2778%	-1.27	0.9216	6.1110%	5.7026%	-1.19	0.9627
Desulfovibrionaceae	1.1578%	1.5727%	2.76	0.9216	0.9331%	1.6329%	2.08	0.9627
Rikenellaceae	1.1679%	0.6915%	-1.44	0.9216	1.5924%	1.0926%	-2.06	0.9627
Lactobacillaceae	1.4827%	1.5694%	1.33	0.9216	0.4334%	0.6920%	2.14	0.9627
Marinifilaceae	0.7895%	0.3671%	-1.58	0.9216	2.2631%	0.7257%	-4.66	0.9627
Saccharimonadaceae	0.6012%	1.7296%	3.11	0.8298	0.5001%	0.8213%	1.60	0.9627
Bacteroidaceae	0.2990%	2.3036%	9.70	0.0004	0.4919%	0.5571%	1.12	0.9627
Akkermansiaceae	1.0615%	0.4136%	-2.40	0.9216	1.1690%	0.5407%	-2.82	0.9627
Tannerellaceae	0.3714%	0.6444%	2.50	0.9216	0.6631%	0.8877%	1.07	0.9627
Helicobacteraceae	0.4983%	0.8571%	1.90	0.9216	0.4878%	0.6054%	1.41	0.9627
uncultured bacterium-04	0.5132%	0.2940%	-1.08	0.9216	0.7858%	0.8166%	-1.31	0.9627
Clostridiales vadinBB60 group	0.1735%	0.4366%	4.04	0.8298	0.6220%	0.5906%	-1.12	0.9627
Deferribacteraceae	0.2077%	0.2569%	1.65	0.9216	0.1443%	0.2222%	-1.06	0.9627
uncultured bacterium-15	0.1142%	0.1897%	1.78	0.9216	0.2677%	0.2472%	1.04	0.9627
Peptococcaceae	0.1431%	0.1948%	3.33	0.9216	0.1327%	0.2231%	1.96	0.9627
Erysipelotrichaceae	0.1483%	0.1499%	1.17	0.9216	0.1846%	0.1368%	-1.33	0.9627
uncultured-04	0.0845%	0.1010%	1.37	0.9216	0.1204%	0.1811%	1.30	0.9627
Family XIII	0.0847%	0.1146%	2.01	0.9216	0.0956%	0.0863%	-1.16	0.9627
Bifidobacteriaceae	0.0757%	0.0773%	1.15	0.9216	0.0394%	0.0678%	1.59	0.9627
Christensenellaceae	0.0347%	0.0556%	1.70	0.9216	0.0790%	0.0840%	-1.32	0.9627
Eggerthellaceae	0.0274%	0.0454%	1.98	0.9216	0.0530%	0.0403%	-1.20	0.9627
Burkholderiaceae	0.0362%	0.0337%	1.07	0.9216	0.0464%	0.0368%	-1.20	0.9627

Table S2c. Differential Abundance (%) Analysis of the 40 most abundant genera detected in groups G2 and G4 at weeks 16 and 18.

Genus	G2 16w Abundance	G2 18w Abundance	G2 18w vs G2 16w - Presence Fold Change	G2 18w vs G2 16w - FDR p-value	G4 16w Abundance	G4 18w Abundance	G4 18w vs G4 16w Log2 Presence Fold Change	G4 18w vs G4 16w FDR p-value
uncultured bacterium-07	24.3150%	26.3560%	1.16	0.9911	23.3357%	25.7377%	-1.13	0.9978
Lachnospiraceae NK4A136 group	11.9858%	8.9817%	-1.10	0.9911	8.3200%	9.6953%	1.35	0.9978
uncultured-15	8.0207%	7.1265%	-1.10	0.9911	6.6331%	6.6948%	1.07	0.9978
uncultured-13	6.0374%	5.8431%	1.00	0.9978	7.3492%	7.6529%	-1.02	0.9978
Ruminococcaceae UCG-014	6.1045%	5.1639%	-1.10	0.9911	9.8599%	5.4716%	-2.29	0.9978
Alloprevotella	3.3918%	3.1316%	-1.04	0.9911	3.7775%	4.1431%	1.01	0.9978
Ruminiclostridium 6	4.4788%	4.1084%	-1.07	0.9911	2.7187%	2.8602%	-1.01	0.9978
Ruminococcus 1	3.6513%	2.1420%	-1.81	0.9911	2.9065%	2.7827%	-1.23	0.9978
[*Eubacterium*] *ruminantium* group	1.0158%	1.8272%	1.94	0.9911	3.2178%	4.1690%	1.45	0.9978
Ruminiclostridium	2.2294%	2.2917%	1.12	0.9911	2.3297%	3.1851%	1.46	0.9978
[*Eubacterium*] *xylanophilum* group	2.3961%	2.1847%	1.02	0.9911	2.4045%	1.6668%	1.01	0.9978
Oscillibacter	1.3509%	2.2993%	1.95	0.9911	2.0529%	1.9485%	1.10	0.9978
Ruminiclostridium 9	1.6275%	1.8203%	1.27	0.9911	1.8435%	2.1052%	1.22	0.9978
Prevotellaceae UCG-001	2.0700%	1.0928%	-1.78	0.9911	2.2529%	1.4857%	-2.35	0.9978
uncultured-26	1.0339%	1.4020%	1.61	0.9911	0.8443%	1.5362%	2.19	0.9978
Coprococcus 2	1.5672%	0.9657%	-1.61	0.9911	1.2512%	0.7669%	-2.29	0.9978
Alistipes	1.0967%	0.6390%	-1.68	0.9911	1.5048%	1.0394%	-1.68	0.9978
Lactobacillus	1.4827%	1.5694%	1.15	0.9911	0.4334%	0.6920%	1.18	0.9978
Muribaculum	1.3713%	1.0157%	-1.41	0.9911	1.1374%	0.4999%	-2.71	0.9978
Ruminococcaceae UCG-003	0.7525%	1.0932%	1.57	0.9911	0.8479%	1.0604%	1.25	0.9978
Candidatus Saccharimonas	0.6012%	1.7296%	3.82	0.7840	0.5001%	0.8213%	1.30	0.9978
Bacteroides	0.2990%	2.3036%	7.44	0.0521	0.4919%	0.5571%	-1.11	0.9978
CAG-352	1.5365%	1.3712%	-1.03	0.9911	0.3707%	0.0222%	-31.09	0.5330
Odoribacter	0.4991%	0.1325%	-3.95	0.9911	2.0493%	0.5303%	-4.11	0.9978
Akkermansia	1.0615%	0.4136%	-2.08	0.9911	1.1690%	0.5407%	-2.87	0.9978
Parabacteroides	0.3714%	0.6435%	1.70	0.9911	0.6631%	0.8872%	1.00	0.9978
Helicobacter	0.4983%	0.8571%	2.20	0.9911	0.4878%	0.6054%	-1.06	0.9978
uncultured bacterium-19	0.5132%	0.2940%	-1.69	0.9911	0.7858%	0.8166%	-1.19	0.9978
Ruminiclostridium 5	0.6283%	0.6372%	1.11	0.9911	0.4984%	0.5390%	1.14	0.9978
[*Eubacterium*] *coprostanoligenes* group	0.4423%	0.9585%	2.57	0.9911	0.5520%	0.3189%	-2.15	0.9978
Ruminococcaceae UCG-010	0.3404%	0.6459%	2.15	0.9911	0.4773%	0.5771%	-1.16	0.9978
Anaerosporobacter	0.1742%	0.4203%	1.97	0.9911	0.6402%	0.7673%	1.41	0.9978
uncultured *Bacteroidales* bacterium	0.4692%	0.3546%	-1.33	0.9911	0.5160%	0.4973%	-1.23	0.9978

uncultured bacterium-21	0.1700%	0.4102%	2.47	0.9911	0.5732%	0.5617%	1.09	0.9978
Ruminococcus 2	0.0055%	1.5057%	255.33	0.0004	0.0068%	0.1351%	15.31	0.9978
Roseburia	0.6153%	0.2701%	-1.57	0.9911	0.2162%	0.5114%	3.45	0.9978
Lachnospiraceae UCG-006	0.4091%	0.3812%	1.20	0.9911	0.3122%	0.3179%	1.17	0.9978
Ruminococcaceae NK4A214 group	0.2132%	0.2393%	1.12	0.9911	0.3393%	0.3944%	1.31	0.9978
GCA-900066575	0.2532%	0.2733%	1.08	0.9911	0.3283%	0.3314%	-1.06	0.9978
Ruminococcaceae UCG-013	0.1597%	0.4761%	3.03	0.9911	0.2337%	0.2669%	1.36	0.9978
