# Supplementary material for: Effect of Clindamycin on Intestinal Microbiome and Miltefosine Pharmacology in Hamsters Infected with Leishmania infantum
Source: Antibiotics (Basel). 2023 Feb 9;12(2):362. doi: 10.3390/antibiotics12020362 (PMC9952363; doi:10.3390/antibiotics12020362)
Supplement: Supplementary file 1 [file antibiotics-12-00362-s001.zip › 4. SUPPLEMENTARY INFORMATION 4 Tables S3a, S3b and S3c (rev).pdf]

SUPPLEMENTARY INFORMATION 4. Table S3a; Table S3b and Table S3c

Table S3a: Differential Abundance (%) Analysis of the phyla detected in groups G4 and G5 at week 18 and PF.

Phylum	G4 18w Abundance	G5 18w Abundance	G5 18w vs G4 18w - Abundance Fold Change	G5 18w vs G4 18w - FDR p-value	G4 PF Abundance	G5 PF Abundance	G5 PF vs G4 PF Abundance Fold change	G5 PFvs G4 PF PF-FDR p-value
Firmicutes	58.7055%	44.2926%	-1.26	0.6159	54.8841%	50.7090%	-1.11	0.9297
Bacteroidetes	35.7798%	47.3925%	1.41	0.1666	39.8885%	43.2804%	1.03	0.9593
Proteobacteria	1.9341%	3.1373%	1.93	0.3520	1.3500%	2.1649%	1.71	0.5945
Cyanobacteria	0.8523%	0.3840%	-2.29	0.0598	0.5490%	0.6104%	1.01	0.9881
Patescibacteria	0.8224%	0.5218%	-1.45	0.6159	0.5922%	0.6891%	1.06	0.9593
Epsilonbacteraeota	0.6056%	0.8948%	1.49	0.6159	0.6024%	0.6267%	-1.13	0.9481
Verrucomicrobia	0.5480%	2.5723%	7.15	0.0432	1.3128%	0.9728%	-1.80	0.7925
Tenericutes	0.3195%	0.4443%	1.30	0.7671	0.3204%	0.1797%	-2.10	0.4961
Deferribacteres	0.2222%	0.0240%	-7.53	0.0072	0.2322%	0.1784%	-1.17	0.9481
Actinobacteria	0.1484%	0.1286%	-1.07	0.9848	0.1934%	0.2472%	1.20	0.9297
Elusimicrobia	0.0497%	0.0199%	-2.51	0.6159	0.0494%	0.3207%	4.51	0.4961
Chloroflexi	0.0033%	0.0000%	-26.28	0.1527	0.0071%	0.0026%	-2.51	0.5945
Fusobacteria	0.0031%	0.1846%	60.55	0.0000	0.0031%	0.0023%	-1.36	0.9297
Planctomycetes	0.0031%	0.0000%	-25.34	0.1527	0.0067%	0.0031%	-1.79	0.7925
Lentisphaerae	0.0015%	0.0019%	1.26	0.9612	0.0013%	0.0044%	3.29	0.4961
Euryarchaeota	0.0004%	0.0014%	3.18	0.3520	0.0000%	0.0047%	37.41	0.1201
Spirochaetes	0.0004%	0.0000%	-4.01	0.6159	0.0019%	0.0010%	-1.66	0.8271
Deinococcus-Thermus	0.0004%	0.0000%	-3.97	0.6159	0.0023%	0.0013%	-1.76	0.7925
WPS-2	0.0002%	0.0000%	-2.47	0.7671	0.0006%	0.0003%	-1.88	0.8271
Gemmatimonadetes	0.0002%	0.0000%	-2.47	0.7671	0.0017%	0.0000%	-15.38	0.4961
BRC1	0.0000%	0.0000%	0.05	0.9848	0.0000%	0.0005%	5.04	0.5945
Chrysiogenetes	0.0000%	0.0000%	0.05	0.9848	0.0000%	0.0005%	5.04	0.5945
Synergistetes	0.0000%	0.0000%	0.05	0.9848	0.0008%	0.0000%	-7.42	0.5945

Table S3b. Differential Abundance (%) Analysis of the families with an abundance >0.01% detected in groups G4 and G5 at week 18 and PF.

Family	G4 18w Abundance	G5 18w Abundance	G5 18w vs G4 18w - Abundance Fold Change	G5 18w vs G4 18w - FDR p-value	G4 PF Abundance	G5 PF Abundance	G5 PF vs G4 PF Abundance Fold change	G5 PFvs G4 PF PF-FDR p-value
Ruminococcaceae	29.1282%	19.3249%	-1.84	0.4847	29.1878%	25.9780%	-1.29	0.8575
Lachnospiraceae	27.7307%	23.9904%	-1.62	0.8771	23.1925%	21.7134%	-1.16	0.9618
Muribaculaceae	26.7692%	23.9232%	1.00	0.9920	31.4890%	31.7463%	-1.25	0.8455
Prevotellaceae	5.7026%	5.0367%	1.16	0.8771	5.5797%	3.1548%	-1.92	0.8288
Desulfovibrionaceae	1.6329%	0.6540%	-3.53	0.2844	1.1082%	1.6712%	1.70	0.8455
Rikenellaceae	1.0926%	0.9589%	1.13	0.8771	0.9067%	0.9596%	-1.05	0.9618
Tannerellaceae	0.8877%	6.5018%	10.10	0.0000	0.8427%	2.6404%	2.15	0.8288
Saccharimonadaceae	0.8213%	0.5216%	-1.41	0.8771	0.5880%	0.6881%	1.31	0.8827
uncultured bacterium-04	0.8166%	0.3790%	-1.60	0.7239	0.4904%	0.5405%	-1.42	0.8379
Marinifilaceae	0.7257%	0.2026%	-2.41	0.5372	0.6233%	0.3016%	-2.78	0.8288
Lactobacillaceae	0.6920%	0.2268%	-2.78	0.5611	1.7371%	1.9552%	-1.26	0.9618
Helicobacteraceae	0.6054%	0.8948%	1.59	0.8771	0.6018%	0.6252%	-1.04	0.9815
Clostridiales vadinBB60 group	0.5906%	0.0970%	-5.47	0.0253	0.2388%	0.4543%	1.25	0.9618
Bacteroidaceae	0.5571%	10.7110%	24.10	0.0000	0.3675%	4.4214%	8.01	0.0002
Akkermansiaceae	0.5407%	2.5720%	8.16	0.0354	1.2987%	0.9640%	-1.95	0.8295
uncultured bacterium-15	0.2472%	0.3394%	1.73	0.7831	0.2370%	0.1300%	-2.35	0.8288
Peptococcaceae	0.2231%	0.0749%	-3.80	0.2193	0.1243%	0.1315%	-1.01	0.9947
Deferribacteraceae	0.2222%	0.0240%	-6.61	0.0315	0.2322%	0.1784%	-1.71	0.8455
uncultured-04	0.1811%	1.6086%	14.07	0.0000	0.0471%	0.3194%	4.72	0.2816
Erysipelotrichaceae	0.1368%	0.1971%	2.16	0.5307	0.1746%	0.1890%	-1.22	0.9618
Family XIII	0.0863%	0.1368%	1.22	0.8771	0.0838%	0.1406%	1.42	0.8575
Christensenellaceae	0.0840%	0.1455%	2.93	0.0934	0.0649%	0.0924%	-1.00	0.9947
Bifidobacteriaceae	0.0678%	0.0430%	-1.17	0.8771	0.1110%	0.1007%	-1.71	0.8295
Elusimicrobiaceae	0.0497%	0.0199%	-1.12	0.9036	0.0490%	0.3207%	4.26	0.8288
Eggerthellaceae	0.0403%	0.0362%	1.09	0.8771	0.0486%	0.1100%	2.04	0.8288
Burkholderiaceae	0.0368%	0.3552%	12.78	0.0000	0.0505%	0.0456%	-1.32	0.8827
unidentified	0.0355%	0.0014%	-16.64	0.0005	0.0204%	0.0303%	1.26	0.9618
Flavobacteriaceae	0.0283%	0.0581%	2.25	0.6572	0.0486%	0.0337%	-1.69	0.8455
Enterobacteriaceae	0.0254%	0.5180%	37.14	0.0013	0.0102%	0.0484%	3.54	0.8295
Atopobiaceae	0.0249%	0.0444%	2.65	0.5611	0.0096%	0.0189%	1.35	0.9618
Anaeroplasmataceae	0.0197%	0.0000%	-105.24	0.1283	0.0131%	0.0000%	-184.54	0.3017
uncultured rumen bacterium-01	0.0156%	0.0007%	-8.17	0.3131	0.0254%	0.0443%	1.52	0.9500
Rhodobacteraceae	0.0152%	0.0002%	-25.96	0.0994	0.0336%	0.0217%	-2.48	0.8295
Defluviitaleaceae	0.0116%	0.0768%	10.05	0.0026	0.0171%	0.0122%	-1.70	0.8455
uncultured rumen bacterium-02	0.0114%	0.0756%	3.66	0.4847	0.0417%	0.0160%	-4.53	0.8288
Xanthomonadaceae	0.0108%	0.0000%	-56.55	0.2158	0.0277%	0.0135%	-3.45	0.8295

Caldicoprobacteraceae	0.0081%	0.0012%	-3.58	0.5132	0.0279%	0.0166%	-2.04	0.8295
Beijerinckiaceae	0.0064%	0.0000%	-31.19	0.3131	0.0146%	0.0088%	-2.63	0.8295
Halomonadaceae	0.0060%	0.0000%	-32.44	0.3131	0.0144%	0.0085%	-2.50	0.8295
Rubritaleaceae	0.0058%	0.0000%	-32.37	0.3131	0.0102%	0.0072%	-1.97	0.8575
uncultured *Lachnospiraceae* bacterium	0.0054%	0.0278%	8.47	0.0727	0.0073%	0.0034%	-2.43	0.8295
Sporolactobacillaceae	0.0037%	0.0000%	-22.48	0.3980	0.0106%	0.0075%	-2.15	0.8455
Fusobacteriaceae	0.0031%	0.1846%	55.28	0.0000	0.0031%	0.0023%	-1.99	0.8455

Table S3c. Differential Abundance (%) Analysis of the genus with significant abundance changes (FDR p>0.05) detected in groups G4 and G5 at week 18 and PF.

**Genus**	**G4 18w Abundance**	**G5 18w Abundance**	**G5 18w vs G4 18w - Abundance Fold Change**	**G5 18w vs G4 18w - FDR p-value**	**G4 PF Abundance**	**G5 PF Abundance**	**G5 PF vs G4 PF Abundance Fold change**	**G5 PFvs G4 PF PF-FDR p-value**
[*Eubacterium*] *fissicatena* group	0.0000%	0.1726%	3281.08	0.0000	0.0000%	0.0000%	-1.06	0.9944
Morganella	0.0000%	0.1370%	2660.27	0.0000	0.0004%	0.0005%	1.30	0.9944
Sutterella	0.0000%	0.0468%	880.64	0.0002	0.0002%	0.0003%	1.10	0.9944
Erysipelatoclostridium	0.0004%	0.0490%	191.33	0.0001	0.0002%	0.0481%	163.59	0.0031
Parasutterella	0.0077%	0.2892%	80.26	0.0000	0.0131%	0.0122%	1.05	0.9944
Ruminococcus 2	0.1351%	5.3202%	52.32	0.0019	0.0150%	0.0080%	-1.78	0.9944
Azospirillum sp. 47_25	0.0100%	0.1915%	50.61	0.0001	0.0063%	0.0660%	12.58	0.1006
Escherichia-Shigella	0.0252%	0.3759%	40.22	0.0021	0.0098%	0.0456%	4.97	0.9944
Eubacterium	0.0002%	0.0089%	34.73	0.0165	0.0002%	0.0021%	7.48	0.9944
Proteus	0.0002%	0.0050%	34.45	0.0181	0.0000%	0.0023%	22.05	0.7717
Fusobacterium	0.0031%	0.1846%	33.68	0.0000	0.0031%	0.0023%	-1.37	0.9944
ASF356	0.0008%	0.0211%	32.91	0.0019	0.0008%	0.0008%	-1.16	0.9944
Bacteroides	0.5571%	10.7110%	29.50	0.0000	0.3675%	4.4214%	14.04	0.0000
Bilophila	0.0225%	0.2011%	21.09	0.0004	0.0138%	0.0764%	6.87	0.1764
CAG-352	0.0222%	0.6521%	20.59	0.0483	0.0169%	0.4406%	26.18	0.0923
gut metagenome-03	0.1607%	1.3943%	18.80	0.0000	0.0373%	0.2203%	6.55	0.0825
Marvinbryantia	0.0027%	0.0557%	15.80	0.0052	0.0036%	0.0463%	14.42	0.0416
Blautia	0.0343%	0.3615%	14.95	0.0001	0.0336%	0.0378%	1.38	0.9944
Parabacteroides	0.8872%	6.5018%	13.94	0.0000	0.8427%	2.6402%	3.31	0.2974
[*Eubacterium*] *nodatum* group	0.0037%	0.0665%	12.96	0.0039	0.0029%	0.0101%	3.68	0.9929
Akkermansia	0.5407%	2.5720%	12.08	0.0088	1.2987%	0.9640%	-1.73	0.9944
Defluviitaleaceae UCG-011	0.0116%	0.0768%	6.99	0.0411	0.0171%	0.0122%	-1.43	0.9944
Ruminiclostridium 6	2.8602%	0.6742%	-3.85	0.0286	2.9644%	1.6275%	-1.76	0.9944
Ruminococcaceae UCG-014	5.4716%	0.8043%	-4.13	0.0420	8.7869%	6.0254%	-1.70	0.9944
uncultured bacterium-21	0.5617%	0.0826%	-4.78	0.0361	0.2299%	0.3953%	1.45	0.9944
Ruminococcaceae NK4A214 group	0.3944%	0.0706%	-5.16	0.0035	0.2342%	0.1662%	-1.61	0.9944
Mucispirillum	0.2222%	0.0240%	-5.70	0.0482	0.2322%	0.1784%	-1.36	0.9944
Lachnospiraceae UCG-008	0.0570%	0.0089%	-6.94	0.0040	0.0330%	0.0150%	-2.30	0.9944
Peptococcus	0.0347%	0.0041%	-7.09	0.0031	0.0142%	0.0181%	1.14	0.9944
Anaerovorax	0.0148%	0.0014%	-7.47	0.0132	0.0204%	0.0357%	1.54	0.9944
Lachnospiraceae UCG-006	0.3179%	0.0322%	-8.33	0.0001	0.3244%	0.1926%	-1.54	0.9944
Lachnospiraceae UCG-002	0.1118%	0.0106%	-10.82	0.0053	0.1275%	0.0567%	-2.08	0.9944

A2	0.0189%	0.0022%	-12.36	0.0198	0.0250%	0.0016%	-15.69	0.0598
Rikenellaceae RC9 gut group	0.0493%	0.0024%	-13.19	0.0005	0.0751%	0.0137%	-5.89	0.0598
unidentified	0.0355%	0.0014%	-13.22	0.0032	0.0204%	0.0303%	1.13	0.9944
[*Ruminococcus*] *torques* group	0.0131%	0.0007%	-13.68	0.0377	0.0192%	0.0057%	-3.77	0.9944
[*Eubacterium*] *ruminantium* group	4.1690%	0.2417%	-16.38	0.0128	4.7466%	1.9847%	-2.39	0.9944
Odoribacter	0.5303%	0.0101%	-20.96	0.0152	0.4259%	0.0753%	-5.03	0.9944
Oscillospira	0.0066%	0.0002%	-23.81	0.0232	0.0038%	0.0044%	1.06	0.9944
Moryella	0.0073%	0.0002%	-25.49	0.0454	0.0069%	0.0016%	-3.80	0.9944
Lachnospiraceae UCG-004	0.4825%	0.0048%	-57.92	0.0163	0.1706%	0.0062%	-34.84	0.1344
